# Autochthonous, zoonotic *Onchocerca lupi* in a South Texas dog, United States

**DOI:** 10.1186/s13071-021-04707-4

**Published:** 2021-04-15

**Authors:** Guilherme G. Verocai, Caroline Sobotyk, Allegra Lamison, Mindy M. Borst, Erin E. Edwards

**Affiliations:** 1 Department of Veterinary Pathobiology, College of Veterinary Medicine and Biological Sciences, Texas A&M University, College Station, TX 77843 USA; 2 North 10th Street Animal Hospital, McAllen, TX USA; 3 Texas A&M Veterinary Medical Diagnostic Laboratory, College Station, TX USA

**Keywords:** Filarioidea, Ocular onchocercosis, Vector-borne diseases, Zoonotic onchocerciasis

## Abstract

**Background:**

*Onchocerca lupi* is an emerging, zoonotic filarioid nematode associated with ocular disease in companion animals in North America and the Old World. The areas where this parasite is assumed to be endemic in the USA comprise southwestern states. Thus far, all cases reported outside of the southwest are associated with travel or animal movement.

**Methods:**

An 11-year-old, castrated male Pitbull dog from McAllen, Hidalgo County, southern Texas, with no travel history, was diagnosed with a perforating corneal ulceration of the right eye. Enucleation was performed and tissues submitted for histopathology.

**Results:**

Histologically, sections of two filarioid nematodes were observed. DNA was extracted from formalin-fixed paraffin-embedded tissue using a commercial kit. We performed PCR targeting the *cox1* gene of the mitochondrial DNA, followed by sequencing and phylogenetic analysis. Altogether, these results confirmed the identification of the nematode specimens as *O. lupi*, phylogenetically belonging to haplotype 1.

**Conclusion:**

We report the first autochthonous case of *O. lupi* in a dog from Hidalgo County, southern Texas, USA. Our finding suggests Texas as an additional state where this zoonotic nematode is endemic. Further investigations are required to understand the epidemiology of this parasite along the USA/Mexico border.

**Graphic abstract:**

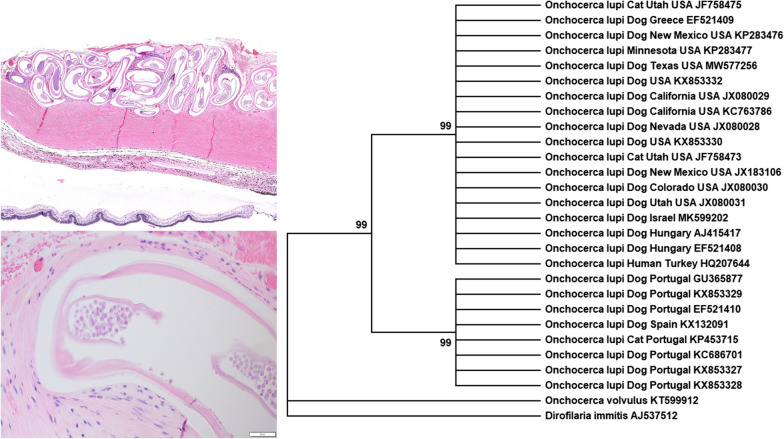

## Background

*Onchocerca lupi* (Nematoda: Onchocercidae) is an emerging, zoonotic parasite in North America and areas of the Old World [1–4]. Infection in dogs and cats is usually associated with episcleral and periocular tissues causing minor to severe ocular lesions, including conjunctivitis, third eyelid prolapse, exophthalmos, uveitis, and retinal detachment [2, 5, 6]. Clinical management and treatment of ocular onchocercosis can be challenging and may involve surgical removal of nodules or require enucleation [5]. To date, no medical treatment seems to be 100% effective against *O. lupi* adults, including regimens of macrocyclic lactones, melarsomine, and the combination of both with tetracyclines. Recurrence of ocular nodules are not uncommon [5, 6]. Oxfendazole seems to impact the number of microfilariae in skin [7].

The presentation of zoonotic onchocerciasis by *O. lupi* in humans may be variable according to the site of infection. To date, there have been seven confirmed zoonotic onchocerciasis cases by *O. lupi* in the USA. Six of these cases involved children [1, 3]. An additional medical concern is that three of these cases were associated with nodules at the cervical spinal cord; two of these cases had gravid adult female specimens, suggesting that these were patent infections. These North American human *O. lupi* infections have been reported from Arizona, New Mexico, and Texas. While this parasite is considered endemic in Arizona and New Mexico, from where multiple cases have been reported in companion animals, humans and wildlife [3, 5, 8], the origin of the single human case in southern Texas remains uncertain. Thus far, all canine cases in North America reported outside of the southwestern USA (e.g., Minnesota, Florida, New York) have been associated with travel [2, 9, 10]. Similarly, some European cases in dogs and humans have been linked to translocation or travel to endemic regions of the Mediterranean [11–13].

## Materials and methods

In August 2020, an 11-year-old, castrated male Pitbull dog from McAllen, Hidalgo County, southern Texas, was presented to the veterinarian with a history of ocular irritation for approximately 2–3 weeks. It was diagnosed with a perforating corneal ulceration of the right eye. The dog was born in McAllen and had never left the Rio Grande Valley, Texas. The animal tested antigen-positive for heartworm, *Dirofilaria immitis*, during pre-anesthetic work-up, despite somewhat compliant chemoprophylaxis with an ivermectin-based monthly product. An enucleation was performed, and the eye was fixed in 10% neutral buffered formalin and submitted to the Texas A&M Veterinary Medical Diagnostic Laboratory for histological processing and examination.

Genomic DNA was extracted using a Qiagen FFPE Tissue DNA extraction kit (Qiagen, USA) according to the manufacturer’s instructions. Polymerase chain reaction (PCR) was performed in 25 µl reactions containing 0.25 μM of each primer, 1 × GoTaq® Green Master Mix (Promega Corporation, Madison, WI, USA), and 2.5 µl of DNA template. The cytochrome oxidase c subunit 1 (*cox1*) gene was amplified using the forward primer COIintF: 5′-TGA TTG GTG GTT TTG GTA A-3′ and reverse primer COIintR: 5′-ATA AGT ACG AGT ATC AAT ATC- 3′ [2, 14, 15]. The cycling conditions included an initial denaturation step at 95 ℃ for 2 min, followed by 40 cycles at 95 °C for 45 s, 52 ℃ at 45 s, and 72 ℃ for 90 s, and a final extension step at 72 °C for 5 min. PCR products were purified using E.Z.N.A.® Cycle Pure Kit (Omega Bio-tek, Norcross, GA, USA) according to the manufacturer’s instructions, followed by Sanger sequencing. Phylogenetic analysis was performed in MEGA X using the maximum likelihood method and general time reversible, gamma distributed as the best fit model [16].

## Results

Our report represents the first unequivocally autochthonous case of *O. lupi*, an agent of zoonotic onchocerciasis, in Texas, near the USA/Mexico border, based on integrated histopathological, parasitological, and molecular data.

**Fig. 1:**
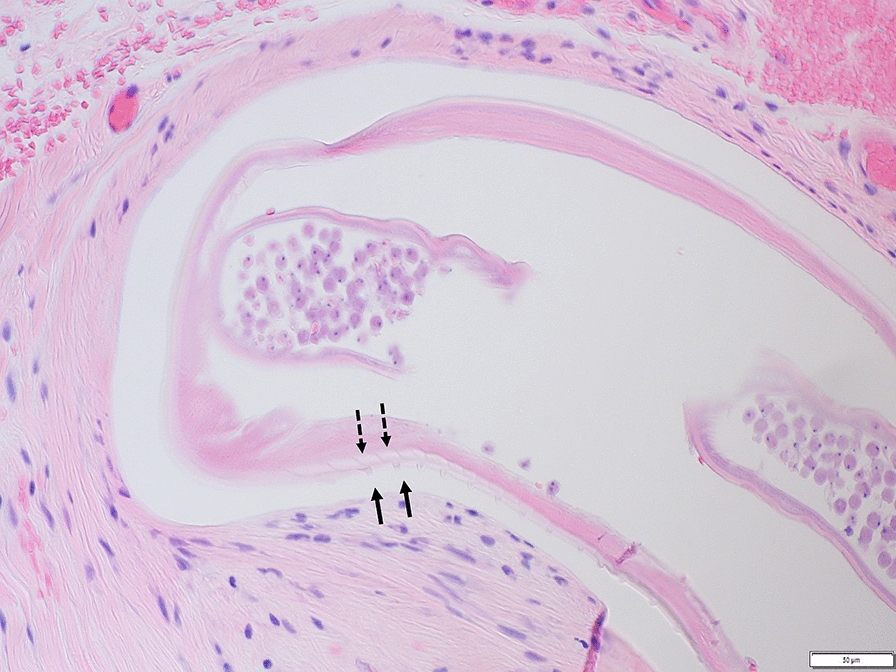
Histological section of the dog’s ocular tissue evidencing an *Onchocerca lupi* specimen. This gravid female specimen was embedded in the episclera of a dog from South Texas. The cuticular pattern of two inner striae (dashed arrows) within the space between two outer cuticular ridges (solid arrows) aided in the species-level identification. (H&E, 40×, bar = 50 µm)

The patterns of cuticular ridges of two inner striae within the space between two outer cuticular ridges observed on the specimens were morphologically consistent with *O. lupi* (Fig. 1). Histologically, the most significant finding was the presence of corneal perforation with severe keratitis and anterior iris synechiae. Additionally, two long, filarioid nematodes were discovered embedded in the episcleral tissues. One of these parasites was degenerated and surrounded by granulomatous inflammation, while the other was intact and lacked surrounding inflammation. This specimen was a gravid female nematode, suggesting a patent infection. Most likely, the dog was co-infected with heartworm, *D. immitis*, as a recent study, has shown that *O. lupi* infections are unlikely to generate false-positive results in commercial heartworm antigen tests [17].

**Fig. 2:**
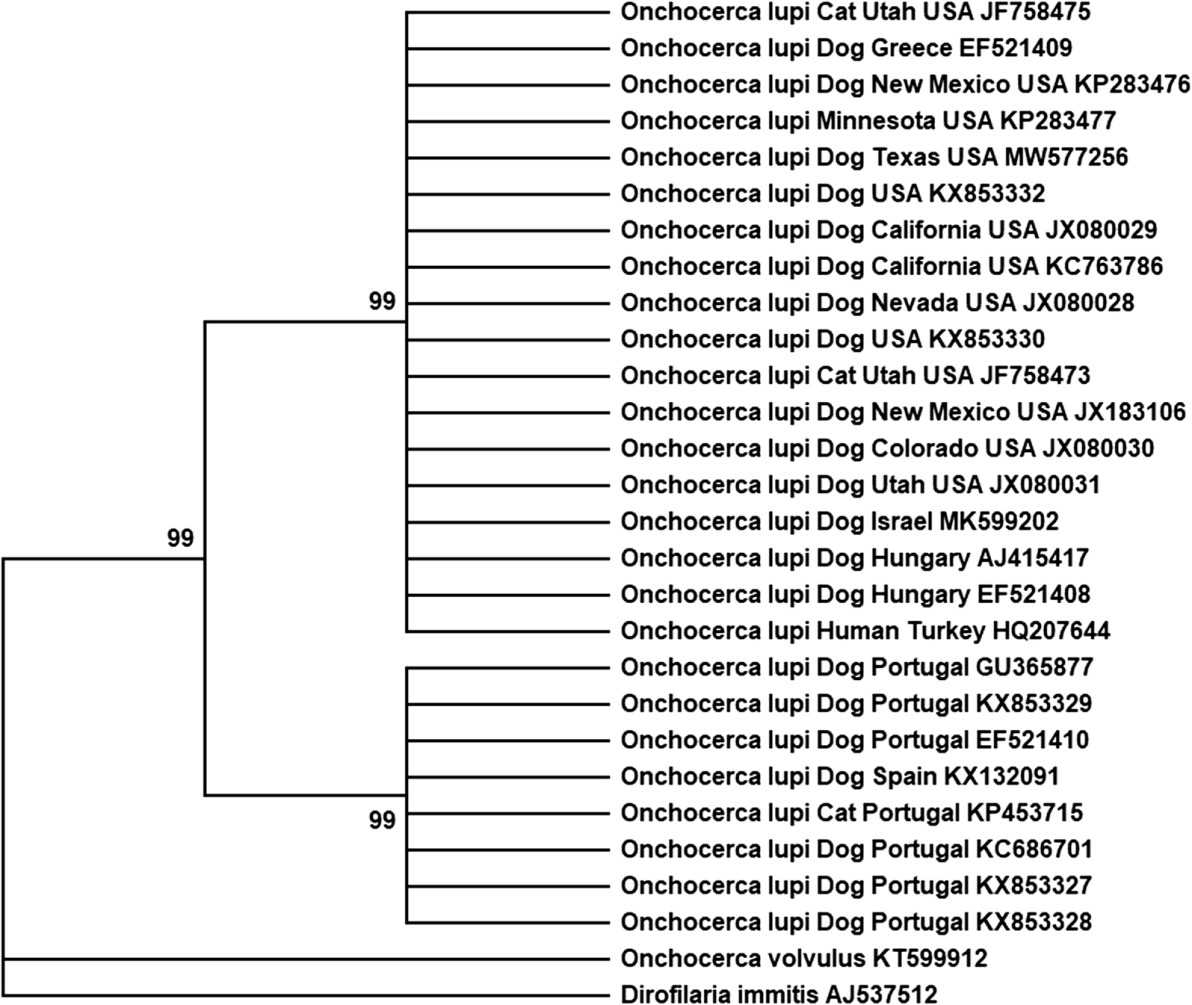
Phylogenetic tree depicting relationships of the canine *Onchocerca lupi* isolate from South Texas, USA. Analysis was performed using the maximum likelihood method (1000 bootstrap replicates) and included other species of *Onchocerca*, and *Dirofilaria immitis* as outgroup. Nodes with < 90% support were condensed

The generated *cox1* sequence was accessioned in GenBank (MW577256), and showed 99.9–100% maximum identity with *O. lupi* sequences available in GenBank. Phylogenetic analysis clustered the Texas isolate with all previous isolates from North America, and some European isolates, belonging to *O. lupi* “genotype 1” (99% bootstrap support; Fig. 2) [18].

## Discussion

This case of canine ocular onchocercosis seems to be the first unequivocal, autochthonous report from the state of Texas. Similar to other clinical reports of *O. lupi* infections in dogs, it is postulated that the parasite could have caused exophthalmos with secondary traumatic corneal ulceration and subsequent perforation [2, 5].

Zoonotic onchocerciasis has been previously reported from a 10-year-old boy from Mission, Texas, a city also located in Hidalgo County. However, the boy had traveled to South Dakota and slept in tents and cabins in New Mexico and Colorado, reported fishing in fresh water lakes near home, and had a pet dog with history of conjunctivitis and an eye lesion of unknown etiology [3]. Altogether, the present canine case may suggest that this human infection could have also been acquired in South Texas.

It is necessary to better understand the epidemiology of *O. lupi* in this newly recognized endemic area by screening dogs and cats from shelters using classical and molecular methods. In addition to companion animals and humans, coyotes (*Canis latrans*) were reported infected and may serve as wild reservoirs in the western USA [8] and may also contribute to the epidemiology of *O. lupi* in southern Texas and neighboring Mexico. Other wild carnivores that are known or postulated to be *O. lupi* hosts, such as wolves (*Canis lupus*) and certain foxes (red fox, *Vulpes vulpes*; swift fox, *Vulpes velox*; kit fox, *Vulpes macrotis*), are not present in southern Texas. However, the widely distributed gray fox (*Urocyon cinereoargenteus*) is reported in this region and across areas where *O. lupi* is endemic in North America [19] and therefore should be assessed as a potential reservoir host.

Regarding the dipteran vectors, black flies (Simuliidae) have been considered the putative intermediate host for *O. lupi* [20]; however, biological confirmation remains necessary. Various mammalophilic black fly species of the genus *Simulium* have been reported from South Texas, USA, especially within the Rio Grande Valley and the neighboring Mexican state of Tamaulipas and up the Rio Grande, along the USA/Mexico border [21, 22]. Among these are *Simulium tribulatum*, postulated as a putative vector of *O. lupi*, and other *Onchocerca* species in southern California [20, 23], *Simulium mediovittatum*, and *Simulium meridionale*. Ideally, however, xenomonitoring in Texas could include screening of dipterans other than simuliids, such as biting midges (Ceratopogonidae: *Culicoides*) and sand flies (Psychodidae: Phlebotominae).

## Conclusion

This unequivocal autochthonous *O. lupi* case in southern Texas suggests that this parasite is endemic in the region, and transmission may occur locally. There is a need for epidemiological surveillance of companion animals, wildlife, and dipteran vectors along the USA/Mexico border. It is important to raise awareness of public health and medical specialists and authorities as humans in this region might be at risk of infection.

## Data Availability

Not applicable.
